# T-Cell Specific Deletion of PTPN23 Alters T-Cell Development and TCRαβ/γδ Composition

**DOI:** 10.3390/cells15181694

**Published:** 2026-09-18

**Authors:** Rocio Sanchez Alvarez, Madita Determann, Luise Linzmeier, Marijn Wilmink, Maria Rae Walker, Anna Bircher, Céline Mamie, Doris Pöhlmann, Marlene Schwarzfischer, Sebastian Zeissig, Claudia Gottier, Silvia Lang, Marianne R. Spalinger, Yasser Morsy, Michael Scharl

**Affiliations:** 1 Department of Gastroenterology and Hepatology, University Hospital Zurich, University of Zurich, Rämistrasse 100, 8091 Zürich, Switzerland; 2 Department of Visceral Surgery and Medicine, University Hospital Bern, 3010 Bern, Switzerland

**Keywords:** PTPN23, T-cell development, TCRγδ

## Abstract

**Highlights:**

**What are the main findings?**

- T-cell–specific deletion of PTPN23 causes a marked reduction in T-cells, accompanied by preferential accumulation of TCRγδ T-cells and enrichment of differentiated effector/memory T-cell subsets.
- Multi-omics and single-cell RNA sequencing reveal alterations in T-cell developmental states, maturation-associated programs, and immune homeostasis following PTPN23 deletion.

**What are the implications of the main findings?**

- These findings identify a novel function of PTPN23 associated with T-cell development, differentiation, and immune homeostasis.
- This study provides a basis for future studies investigating whether PTPN23 contributes to dysregulated T-cell responses, including inflammatory disorders and cancer.

**Abstract:**

T-cells are major therapeutic targets in cancer and immune-mediated diseases. Their development and differentiation require highly dynamic receptor turnover. Protein tyrosine phosphatase non-receptor type 23 (PTPN23) encodes a ubiquitously expressed pseudophosphatase involved in trafficking and internalization of specific cell receptors. Although PTPN23 has essential functions in several cell types, its role in T-cells remains unknown. We therefore investigated the role of PTPN23 in T-cells. We generated transgenic mice lacking PTPN23 specifically in T-cells (PTPN23-CD4-Cre). Flow cytometry, single-cell RNA sequencing (scRNA-seq), and proteomic analysis were performed in lymphoid and mucosal organs. PTPN23-CD4-Cre mice exhibited a significant reduction in total T-cell numbers in lymphoid organs, accompanied by increased proportions and numbers of γδ T-cells and markedly reduced naïve T-cells. Similar findings were made in mucosal organs, with γδ T-cell levels rising as age increased. In vitro functional assays revealed that residual PTPN23-CD4-Cre T-cells display an altered cytokine profile enriched for inflammatory mediators, a finding that was accompanied by proteomic and phosphoproteomic alterations consistent with changes in T-cell differentiation and inflammatory programs. scRNA-seq analysis showed altered representation of thymocytes along a shared developmental trajectory, with enrichment of PTPN23-deficient T-cells at earlier developmental states. Our study identifies PTPN23 as a previously unrecognized player of T-cell development, differentiation, and immune homeostasis.

## 1. Introduction

The T-cell receptor (TCR) is a key component in T-cell responses. The conventional TCR organization, known as TCRαβ, recognizes antigens presented by major histocompatibility complex (MHC) molecules, while the alternative TCR type, TCRγδ, exhibits MHC-unrestricted recognition of antigens [1]. TCRγδ cells are primarily present in mucosa-associated lymphoid tissue (MALT) and epithelial layers, where they perform subset-specific functions, including maintenance of intestinal epithelial integrity [2]. Due to their lack of MHC restriction, they are often classified as innate immune cells, serving as a first line of defense in the intestine [3,4]. In addition, TCRγδ cells also contribute to early adaptive immune response, due to their cytokine and chemokine production. Additionally, TCRγδ cells interact with intestinal epithelial cells and other immune cells, such as monocytes, dendritic cells, neutrophils, and B cells, to coordinate an appropriate immune response [4]. Furthermore, the role of TCRγδ cells in maintaining the symbiotic relationship between the gut microbiota and the immune response has recently been demonstrated [5].

Protein tyrosine phosphatases (PTPs) play an essential role in modulating phosphorylation-dependent pathways, which are particularly active in immune cells [6]. A large fraction of the PTP genes is expressed in lymphocytes, and several members exhibit lymphocyte-specific functions [6]. For instance, PTP non-receptor type 2 (PTPN2) has been implicated in T-cell development; moreover, its inhibition in T-cells has been demonstrated to enhance anti-tumor responses [7,8,9]. PTP non-receptor type 23 (PTPN23) encodes a cytoplasmic pseudophosphatase that participates in the endosomal sorting complex required for transport (ESCRT) machinery and thereby regulates the internalization and trafficking of specific cell receptors, such as epidermal growth factor receptor (EGFR) or MHC class I molecules [10]. PTPN23 is ubiquitously expressed across tissues and cell types, with particularly high expression in the intestinal epithelium. A recent study demonstrated that PTPN23 is essential for intestinal barrier integrity and prevention of fatal bacterial translocation, highlighting a critical role for PTPN23 in intestinal immune homeostasis [11]. Furthermore, PTPN23 has been implicated in several cancer types although a possible role in immune cells has never been studied [12]. Given its expression pattern, its immunomodulatory role in the intestine, and the established relevance of PTPs in immune-regulating cells, we hypothesized that PTPN23 might contribute to T-cell biology, including the differentiation of TCRαβ and TCRγδ composition, and might be an interesting protein for future studies for immune-mediated disorders.

## 2. Materials and Methods

### 2.1. Mice

Mice with loxP-flanked PTPN23 gene (Ptpn23^fl/fl^) were purchased from EUCOMM supplied by INFRAFRONTIER/EMMA (Munich, Germany). Mice carrying the Cre recombinase construct under the CD4 promoter (CD4-Cre) were obtained from The Jackson Laboratory (Bar Harbor, ME, USA). All mice were kept in specific-pathogen-free conditions and provided with food and water ad libitum. The mice were grouped according to genotype into different experimental groups. Experiments were performed on young mice (4–6 weeks) and adult mice (17–28 weeks). Both male and female mice were included in the study. However, sex was not incorporated as an independent variable in the statistical analyses because of the limited sample size within individual genotype-by-age subgroups. To ensure transparency, the sex distribution of the experiments is displayed in Supplementary Figure S1. A maximum of 5 animals were housed in individually ventilated cages type 2 provided with automated watering system and Safe Aspen premium hygiene bedding. The experimental unit was defined as mice. All experiments were performed according to Swiss animal welfare legislation and approved by the local veterinary office (Veterinäramt des Kantons Zürich; ZH018-2025).

### 2.2. Tissue Harvesting

Animals were anesthetized intraperitoneally with a dose of 120 mg/kg body weight using a mixture of 10% Ketamine and 3% Xylazine in NaCl. Mice were sacrificed by cervical dislocation, and transcardiac perfusion was performed with PBS prior to organ collection. Thymus, spleen, liver, lungs, and lamina propria lymphocytes (LPLs) from colon were collected and processed for immune cell isolation. The thymus and spleen were homogenized, passed through a 70 µm strainer using PBS, and centrifuged at 1500 rpm for 10 min at 4 °C. The supernatant was discarded, and the pellets stored at 4 °C. For LPLs, immune cells were isolated as described [11]. Liver and lung were digested using Collagenase Type IV in HBSS with 10% FCS for 45 min at 37 °C. Mechanical homogenization was performed using an 18-gauge needle, followed by filtration through a 70 µm strainer (Supplementary Table S1). For liver, a gradient centrifugation was performed using 30% Percoll in PBS to separate fat from tissue. Finally, for the liver, lungs, and spleen, ammonium-chloride-potassium (ACK) buffer was used to lyse remaining erythrocytes (Supplementary Table S1). All samples were centrifuged at 1500 rpm for 10 min at 4 °C. The supernatant was discarded, and the pellet was stored at 4 °C. The isolated cells were then stained for flow cytometry analysis. Organ harvesting and all downstream experimental procedures were performed in a blinded manner.

### 2.3. Cell Culture

T-cell isolation. Splenocytes were isolated by homogenizing the spleen in 10 mL of RPMI medium supplemented with 10% FCS through a 70 µm strainer (Supplementary Table S2). After 10 min of 1500 rpm centrifugation, the pellet was resuspended to a final concentration of 1 × 10^8^ cells/mL. Isolation of T-cells was performed by using the EasySep™ Mouse T-Cell Isolation Kit (STEMCELL Technologies, Basel, Switzerland; Supplementary Table S2), following the manufacturer’s instructions. To activate the T-cells, isolated T-cells were seeded at a concentration of 2 × 10^5^ cells/well in 96-well coated plates supplemented with RPMI with 10% FCS and kept in a 5% CO_2_ incubator at 37 °C. Previously, the plates were coated with 3 μg/mL anti-CD3 and anti-CD28 in PBS and incubated at 37 °C for 2 h (Supplementary Table S2). β-mercaptoethanol was not added to the culture medium.

### 2.4. Flow Cytometry

First, cells were incubated in PBS containing a viability marker (Supplementary Table S1) for 30 min at 4 °C in the dark. This was followed by incubation with primary antibodies (Supplementary Table S3) for 20 min at 4 °C in the dark, using FACS buffer (2% FCS, 0.01% NaN_3_ in PBS. Sequentially, cells were fixed and permeabilized for intracellular staining (Supplementary Table S3). To restimulate cytokine production prior to staining, cells were incubated for 4 h at 37 °C in RPMI with 10% FCS, phorbol-myristate acetate (PMA), ionomycin, and Brefeldin A (Supplementary Table S1). Samples were resuspended in PBS and passed through a 70 μm filter. Flow cytometry data were acquired using a BD FACSymphony (Becton, Dickinson and Company, Franklin Lakes, NJ, USA) and Cytek Aurora (Cytex Bioscience, Fremont, CA, USA), with FlowJo software (version 10). Data acquired on different flow cytometers were analyzed separately and were not pooled together.

### 2.5. Omics

Single-cell RNA sequencing (scRNA-seq). Thymi were collected from 4–5 weeks-old mice and homogenized, passed through a 70 µm strainer using PBS, and centrifuged at 1500 rpm for 10 min at 4 °C. A cell suspension with a concentration of 2 × 10^3^ cells/ul was prepared and loaded onto the device. Single-cell gene expression libraries were processed with Cell Ranger (10× Genomics) and analyzed in R with Seurat v5 [13]. After merging 10 samples, cells were filtered to retain those with 200–6000 genes, 800–20,000 Unique Molecular Identifier (UMIs), and <20% mitochondrial content. Data were normalized using the LogNormalize method, and the top variable features were used for Principal Component Analysis. Dimensionality reduction and clustering were performed with the Louvain algorithm (resolution 0.4) and visualized via UMAP based on the first 30 principal components. Cell type identities were assigned using SingleR, querying the TILAtlas (mouse v1) for T-cell states, with manual validation against canonical markers [14].

Trajectory Inference and Pseudotime Analysis. To reconstruct developmental trajectories, we employed a multi-tool approach to ensure robustness. Trajectory inference was done on the pooled WT + KO Seurat object rather than separately by genotype. We ran CytoTRACE2 once on pooled raw RNA counts without using genotype labels [15]. We then ran Slingshot once on the pooled UMAP embedding using shared cluster labels and the common root cluster. We retained pseudotime values and lineage weights for all lineages. For genotype comparisons, we used the lineage with the largest total curve weight as the main lineage. We normalized pseudotime once across pooled cells for visualization and sample-level summaries [16]. Gene expression dynamics along the pseudotime axis were modeled using tradeSeq, identifying genes with significant lineage-dependent expression patterns (FDR < 0.05) [17].

Proteomics. T-cells were isolated from spleen as described previously in Section 2.3. FCS was not added to the media to avoid artifacts in the analysis. The cells were washed with PBS twice before freezing the samples. Tandem Mass Tag (TMT) proteomics and phosphoproteomics were performed and analyzed by Functional Genomic Center Zurich (FGCZ).

### 2.6. Statistical Analysis

All statistical analyses and visualizations were conducted using GraphPad Prism v.9 (GraphPad Software). For predefined pairwise comparisons between WT and KO animals within each experimental condition, the two-tailed Mann–Whitney U tests were used because of the small sample sizes and non-normal distribution of the data. No statistical comparisons were performed between different organs, cell subsets, ages, or experimental conditions. Significance levels were set as follows: * *p* < 0.05, ** *p* < 0.01, *** *p* < 0.001, and **** *p* < 0.0001. No symbol indicates no statistically significant difference.

## 3. Results

### 3.1. PTPN23 Depletion Results in T-Cell Reduction

**Figure 1:**
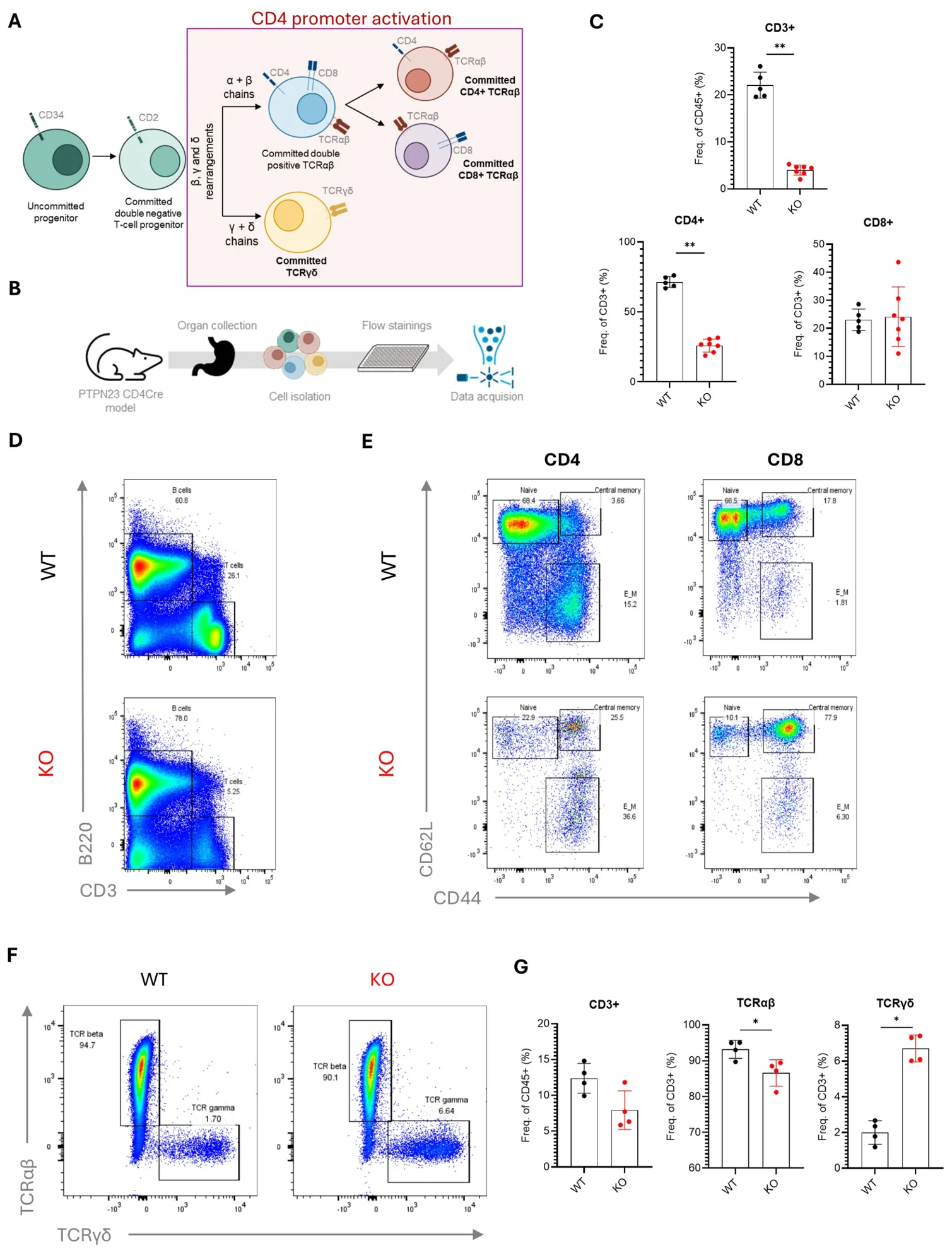
Depletion of PTPN23 in T-cells is characterized by T-cell reduction across lymphoid organs. (**A**) Diagram of T-cell development in the thymus. (**B**) Experimental design of the PTPN23^fl/fl^ CD4-Cre mouse characterization. (**C**) Frequencies of parents of CD45^+^ CD3^+^, CD8^+^ T-cells, CD4^+^ T-cells and subpopulations in splenocytes from WT (black) and KO (red) mice. Two experiments were performed independently (WT, *n* = 4–5 and KO, *n* = 4–5, biological replicates). (**D**) Scatter plots of CD3/CD45, CD4/CD8, and CD62L/CD44 from CD4^+^ T-cells and CD62L/CD44 from CD8^+^ T-cells from splenocytes of WT and KO mice. (**E**) Scatter plots of CD62L/CD44 from CD4^+^ T-cells and CD62L/CD44 from CD8^+^ T-cells from splenocytes of WT and KO mice. (**F**) Scatter plots of TCRαβ/TCRγδ from thymocytes in WT and KO mice. (**G**) Frequencies of parents of CD45^+^ CD3^+^, CD8^+^ T-cells, and CD4^+^ T-cells and subpopulations in thymocytes from WT and KO mice. In panels (**D**–**G**), results shown are from one representative experiment. Three experiments were performed independently (WT, *n* = 4–5 and KO, *n* = 4–5, biological replicates). Data were not pooled due to use of different cytometers. Age of the mice at the termination day was 8–12 weeks. *p*-values were determined by the 2-tailed Mann-Whitney U Test; * *p* < 0.05, ** *p* < 0.01. Unmarked comparisons are not statistically significant. Error bars represent mean ± SD.

To investigate a possible role of PTPN23 in T-cells, we generated a mouse model lacking PTPN23 in all T-cells. In this model, a critical exon of PTPN23 was flanked by loxP sites, which serve as recognition sequences for Cre recombinase. By placing Cre expression under the control of the CD4 promoter, recombination occurs exclusively in T-cells. Because the activation of the CD4 promoter occurs between the double-negative (DN) and the single-positive (SP) selection of CD4^+^ and CD8^+^ T-cells in the thymus (Figure 1A) [18], the knock-out (KO, PTPN23^fl/fl^ CD4-Cre^+/−^) mice lack functional PTPN23 expression in both CD4^+^ and CD8^+^ T-cells.

As a first approach to characterize the model, we isolated splenocytes and sorted them into CD45^+^CD3^+^, CD3^+^CD4^+^, and CD3^+^CD8^+^ populations (Figure 1B). We first confirmed the tissue-specific reduction of PTPN23 expression levels in T-cells from the KO mice in comparison to the wild-type (WT; Supplementary Figure S1). Notably, during sample sorting, we observed a significant decrease in the overall number of T-cells (CD45^+^CD3^+^) in the KO animals (Figure 1C). In initial sorting experiments, a minor relative increase in CD8^+^ T-cell frequency was noted, likely secondary to the marked reduction in CD4^+^ T-cells; however, total absolute CD8^+^ T-cell counts were consistently decreased across all experimental replicates. The marked reduction in T-cell numbers limited the variety of downstream analyses, as for some techniques sufficient numbers of individual T-cell subsets could not be isolated.

To gain a more comprehensive understanding of the immune profile of these mice, we isolated T-cells from lymphoid organs (spleen and thymus) and performed detailed flow cytometry analysis. In the spleen, alongside the depletion of T-cells (Figure 1D), we detected a shift in the T-cell subpopulations. Naïve T-cells (CD4 and CD8) were markedly reduced in KO mice compared to WT (Figure 1E). Conversely, the relative frequencies of effector and central memory T-cells were increased in KO compared to WT mice (Figure 1E). These findings led us to question whether PTPN23 deletion impacts T-cell development, as already demonstrated for other PTPs [6]. Given that T-cell development occurs in the thymus at an early life stage, we next assessed thymocytes using the same experimental approach as outlined above (Figure 1B). We observed a considerable decrease in TCRαβ T-cells, with a proportional increase in TCRγδ T-cells in the KO mice (Figure 1F,G). Taken together, the absence of PTPN23 triggered a significant reduction of the entire T-cell population with an increase in TCRγδ T-cells.

### 3.2. Lack of PTPN23 in T-Cells Causes Age-Dependent Immune Alterations in Lymphoid Organs and Mucosal Tissues

TCRγδ T-cells are primarily localized in mucosal tissues, where they play a key role in innate immune responses. We, therefore, investigated mucosa-associated lymphoid tissue. Given that γδ T-cell generation depends on thymic output, which declines with age due to thymic involution, we compared young mice (4–5 weeks of age), when thymic activity is near its peak, with adult mice (17–28 weeks of age), when thymic involution is established [19].

No difference in weight between WT and KO was observed (Supplementary Figure S2A). Due to thymic involution with age, assessing the thymus size was only feasible in young mice, where no difference was observed between WT and KO mice (Supplementary Figure S2B). However, the normalized spleen weight was significantly reduced in adult KO mice, while no differences were detected between the younger groups (Supplementary Figure S2B). We hypothesize that the reduced spleen size in older KO mice might result from the decrease in T-cell populations observed previously in the flow cytometry data. To extend the immunological characterization of the mice, we next analyzed the immune cell profile of mucosal organs (lung, liver, and LPLs).

**Figure 2:**
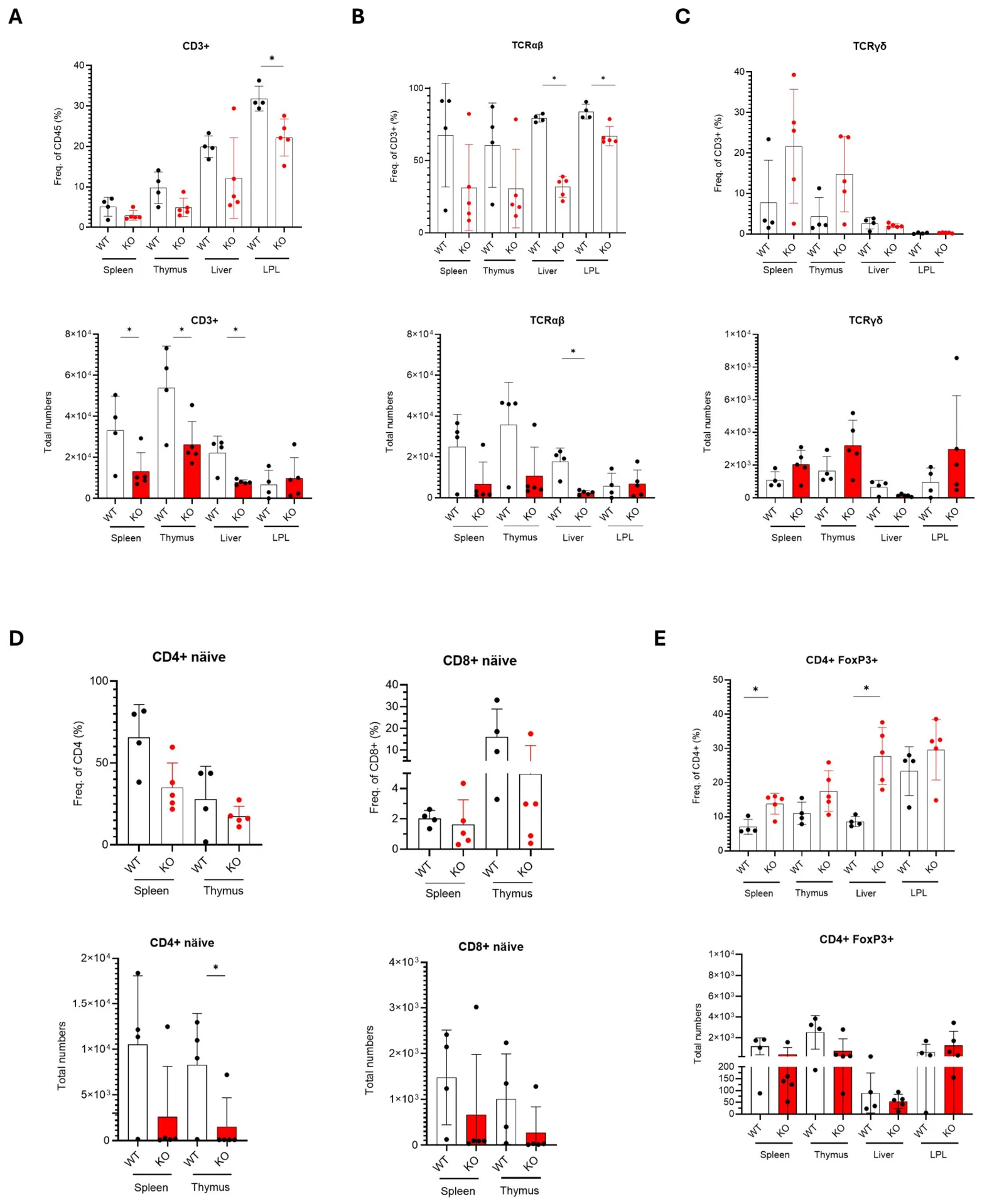
Deletion of PTPN23 in T-cells alters the immune profile in mucosal tissues and lymphoid organs in young mice. (**A**) Frequencies of parents and total numbers of CD3^+^ from spleen, thymus, liver and LPLs in young WT (black) and KO (red) mice. Lung was excluded from the analysis due to the low numbers of isolated immune cells. (**B**) Frequencies of parents and total numbers of TCRαβ^+^ from spleen, thymus, liver, and LPLs. (**C**) Frequencies of parents and total numbers of TCRγδ^+^ from spleen, thymus, liver and LPLs. (**D**) Frequencies of parents and total numbers of naïve CD4^+^ T-cells and naïve CD8+ T-cells from lymphoid organs from young WT and KO mice. (**E**) Frequencies of parents and total numbers of FoxP3 CD4^+^ from spleen, thymus, liver, and LPLs in young WT and KO mice. Two independent experiments were performed (WT, *n* = 4–5 and KO, *n* = 4–5, biological replicates). Data are from one representative experiment. Data were not pooled due to use of different cytometers. *p*-values were determined by the 2-tailed Mann-Whitney U Test; * *p* < 0.05. Unmarked comparisons are not statistically significant. Error bars represent mean ± SD.

In young mice, T-cell depletion in KO animals was confirmed in the lymphoid organs (spleen and thymus), while in mucosal tissues (liver and LPLs) the reduction was less pronounced (Figure 2A). Across the lymphoid organs, KO mice showed a significant decrease in the TCRαβ population together with increased frequencies and total counts of TCRγδ cells (Figure 2B,C). In mucosal tissues, TCRγδ cell numbers were also moderately elevated, particularly in the LPLs (Figure 2C). Analysis of T-cell subsets revealed a marked reduction in naïve T-cells in KO mice within both the CD4^+^ and CD8^+^ populations, accompanied by proportional increases in effector, central memory, and regulatory T-cell (Treg) populations (Figure 2D,E and Supplementary Figure S3A). The increased frequencies of these differentiated subsets likely reflect the reduction of naïve T-cells, resulting in an enrichment of mature T-cell populations among the remaining T-cells.

**Figure 3:**
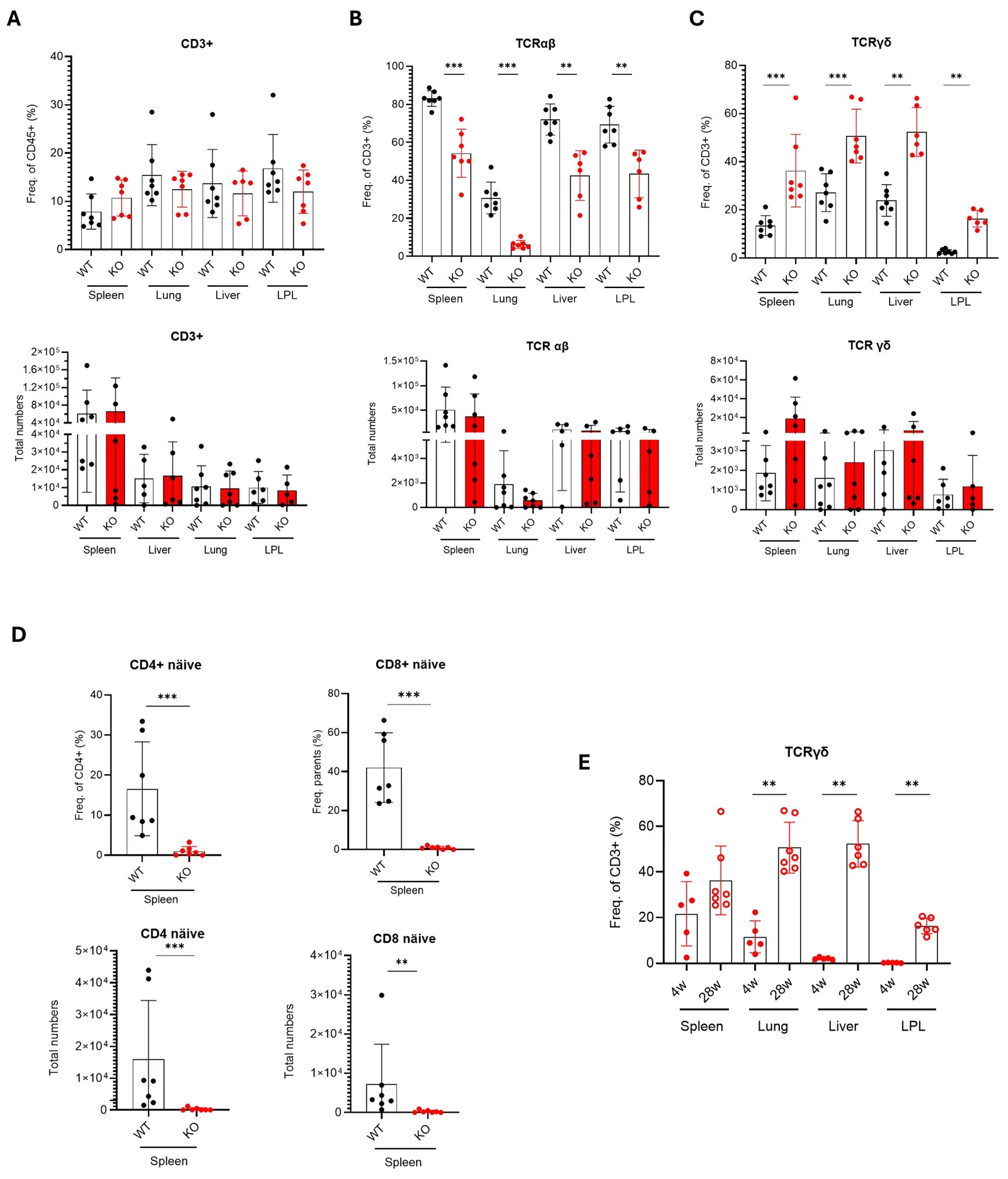
Mice with T-cell-specific PTPN23 deletion show an age-dependent shift in mucosal sites. (**A**) Frequencies of parents and total numbers of CD3^+^ from spleen, lung, liver, and LPLs in adult WT (black) and KO (red) mice. (**B**) Frequencies of parents and total numbers of TCRαβ^+^ from spleen, lung, liver, and LPLs in adult WT and KO mice. (**C**) Frequencies of parents and total numbers of TCRγδ^+^ from spleen, lung, liver, and LPLs in adult WT and KO mice. (**D**) Frequency of parents and total numbers of naïve CD4^+^ T-cells and naïve CD8^+^ T-cells from spleen from adult WT and KO mice. (**E**) Frequency of parents of TCRγδ^+^ from spleen, lung, liver, and LPLs in young and in adult KO animals. Two independent experiments were performed (for each age group WT, *n* = 4–5 and KO, *n* = 4–5, biological replicates). Data from one representative experiment. Data were not pooled due to use of different cytometers. *p*-values were determined by the 2-tailed Mann-Whitney U Test; ** *p* < 0.01, *** *p* < 0.001. Unmarked comparisons are not statistically significant. Error bars represent mean ± SD.

In adult mice, unexpectedly, total CD3^+^ T-cell frequencies were comparable between KO and WT animals in both lymphoid (spleen) and mucosal tissues (lung, liver, and LPL; Figure 3A). However, the TCRγδ cell population remained significantly elevated across all organs while the TCRαβ cells were lower, as observed previously in the young group (Figure 3B,C). We next assessed the T-cell subsets in spleen, where a similar decrease of naïve T-cells and increased frequencies of the effector T-cells were also detected (Figure 3D and Supplementary Figure S3B).

To evaluate the impact of aging on the immune profile of KO mice, we compared TCRγδ frequencies between the young and adult KO cohorts. The older mice showed significantly higher TCRγδ frequencies in mucosal tissues (Figure 3E). These findings imply that aging may mitigate the KO phenotype, particularly the T-cell reduction, in lymphoid organs, while exacerbating it in peripheral tissues, likely due to the migration of the TCRγδ cells.

In conclusion, KO mice exhibit an age-dependent phenotype characterized by a reduction in T-cells, targeting specifically naïve T-cells, and increased TCRγδ cell population.

### 3.3. PTPN23 Deletion in T-Cells Showed Increased Cytokine Production

As we indicated that the absence of PTPN23 in T-cells altered the immune profile under homeostatic conditions, we next investigated whether PTPN23 affects T-cell activation. To that end, we conducted in vitro experiments using isolated T-cells from KO splenocytes.

**Figure 4:**
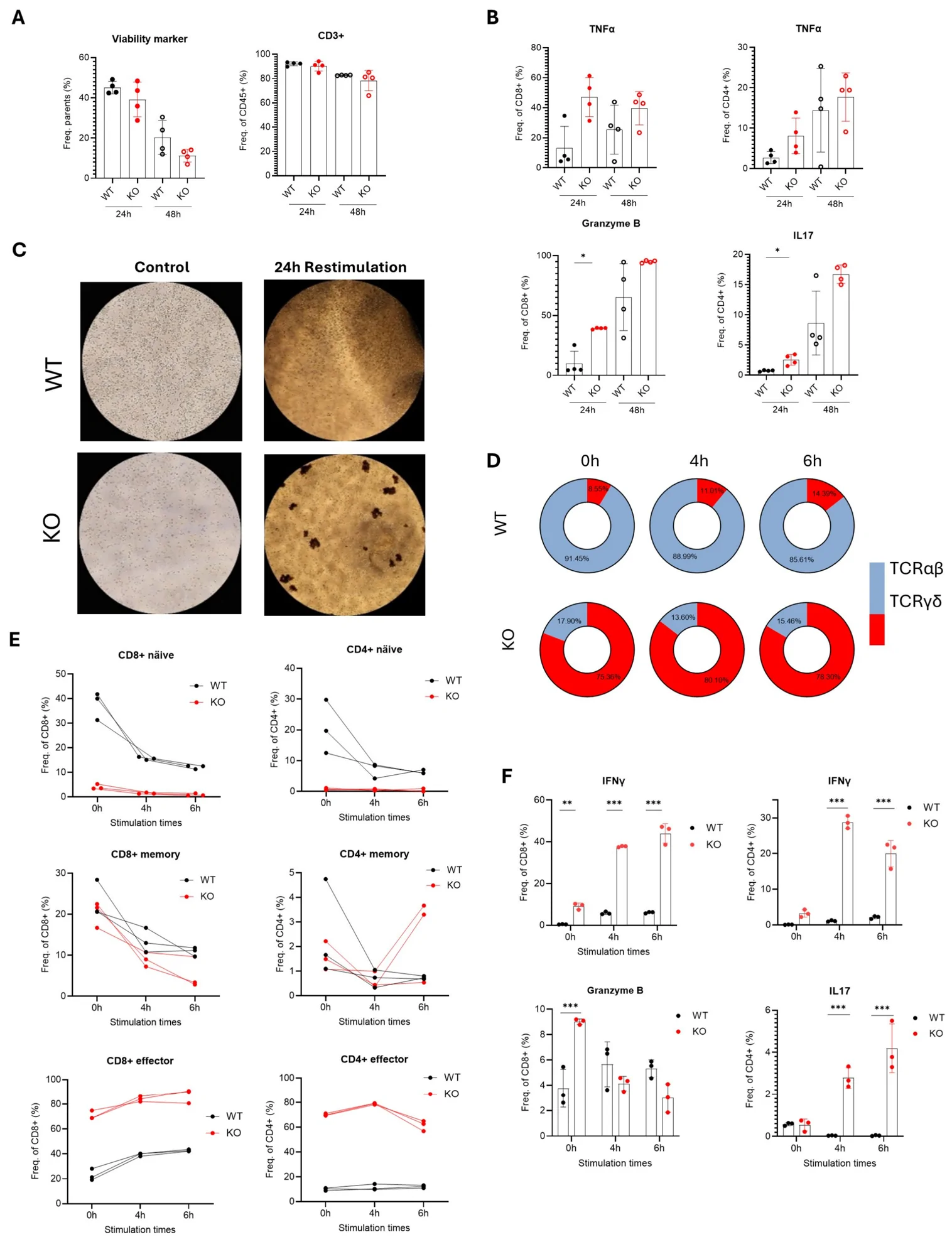
T-cells from KO animals exhibit enhanced production of inflammatory mediators. (**A**) Frequencies of parents of viability marker and CD3^+^ in T-cells from WT (black) and KO (red) mice. (**B**) Frequencies of parents of pro-inflammatory cytokines (TNFα, granzyme B and IL17) in CD4^+^ and CD8^+^ T-cells from WT and KO mice. (**C**) Pictures of isolated T-cells from WT and KO mice at different restimulation times (0 h and 24 h). (**D**) Frequencies of parents TCRαβ^+^ and TCRγδ^+^ T-cells from WT and KO mice at different restimulation times (0 h, 4 h, and 6 h). (**E**) Curve plot representing frequencies of parents of naïve (CD62L^high^ and CD44^low^), central memory (CD62L^high^ and CD44^high^) and effector (CD62L^low^ and CD44^high^) T-cells from WT and KO mice. (**F**) Frequencies of parents of pro-inflammatory cytokines (IFNγ, granzyme B and IL17) in CD4^+^ and CD8^+^ T-cells from WT and KO mice. Two experiments were performed independently (WT, *n* = 4, and KO, *n* = 4, biological replicates). Results shown are from one representative experiment. *p*-values were determined by the 2-tailed Mann-Whitney U Test; * *p* < 0.05, ** *p* < 0.01, *** *p* < 0.001. Unmarked comparisons are not statistically significant. Error bars represent mean ± SD.

To study T-cell activation, we incubated the isolated T-cells in anti-CD3 and anti-CD28 coated plates for 24 h or 48 h, followed by flow cytometry analysis. Initially, we validated the efficiency of the isolation by assessing cell viability and purity. Cell viability was approximately 50% after 24 h of incubation and decreased to around 20% after 48 h, indicating substantial cell loss that may have confounded the interpretation of the observed effects (Figure 4A). The purity of the isolated CD3+ T-cell populations remained high throughout the experiment (Figure 4A). Next, we evaluated the cytokine production (TNFα, IL17 and granzyme B) in TCRαβ^+^ CD4^+^ and TCRαβ^+^ CD8^+^ accordingly after 24 h and 48 h of activation. The measured cytokines showed higher levels after 48 h of activation and, notably, KO cells exhibited higher frequencies of these molecules compared to WT (Figure 4B). In conclusion, PTPN23 deletion in T-cells showed increased levels of TNFα, IL17, and granzyme B upon T-cell activation.

Given these results, we further examined the impact of different T-cell restimulation times on cytokine production in WT and KO cells. As 24 h of activation yielded the highest T-cell viability, we isolated T-cells from both WT and KO mice and activated them for 24 h, and subsequently restimulated them with PMA, ionomycin, and BFA at different time points (0 h, 4 h, 6 h, and 24 h). Notably, after 24 h of restimulation, KO T-cells formed cell clusters, an effect not observed in WT T-cells (Figure 4C). Flow cytometry analysis was conducted after 0 h, 4 h, and 6 h of restimulation. Overall, we did not observe any significant effect associated with longer or shorter restimulation times (Figure 4D–F). However, when comparing WT and KO T-cells, similar effects to the in vivo phenotype were observed. PTPN23-KO T-cells showed significantly increased TCRγδ and reduced TCRαβ populations (Figure 4D). Additionally, KO samples displayed a depletion of naïve T-cells alongside an increase in effector and memory T-cell frequencies (Figure 4E). Typically, upon activation, naïve and memory T-cells differentiate into effector T-cells to build an immune response. In line with this expectation, we observed a reduction in naïve T-cells and a corresponding increase in effector T-cells in both WT and KO samples over the restimulation period. Regarding cytokine production, T-cells from KO mice exhibited elevated levels of IFNγ and IL-17 (Figure 4F). Granzyme B was also increased in KO mice, although this observation was only detected in conditions with no restimulation. However, cytokine differences within the broad TCRαβ CD4^+^ and CD8^+^ compartments may reflect differences in the relative abundance of naïve, effector, and memory subsets and, therefore, cannot be interpreted as evidence of a direct T-cell-intrinsic effect of PTPN23 on cytokine production.

Overall, these findings indicate that PTPN23 deficiency is associated with a relative enrichment of effector and memory T-cell populations and an altered cytokine profile. However, whether PTPN23 deficiency directly enhances cytokine production independently of these compositional changes remains unresolved.

### 3.4. PTPN23-Deficient T-Cell Compartment Exhibits Altered Proteomic and Phosphoproteomic Profiles

**Figure 5:**
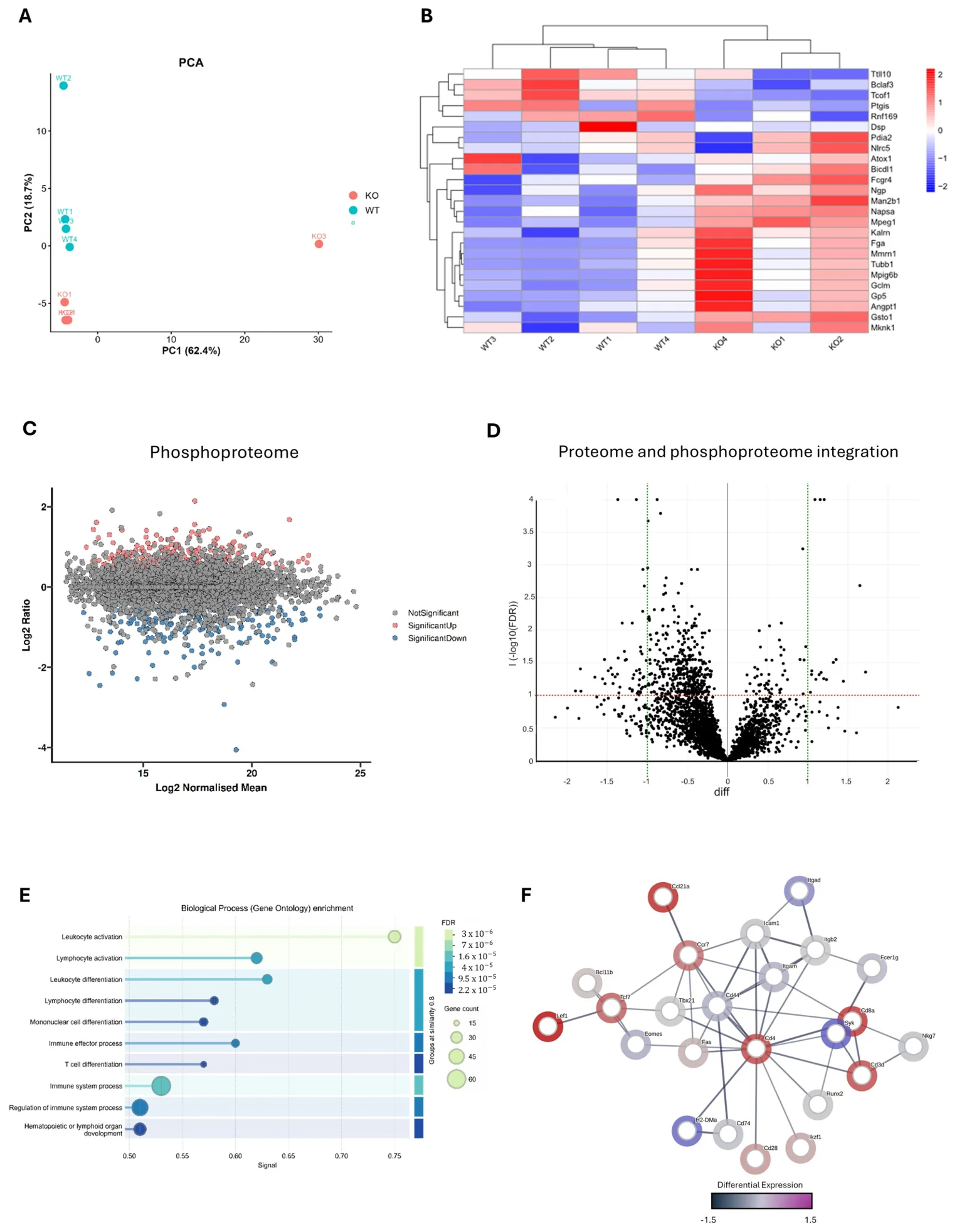
Proteomic and phosphoproteomic profiles of splenocytes differ significantly between KO and control mice. (**A**) Plot of the first and second principal components (PC1 and PC2) of principal component analysis (PCA) from the whole proteome. Normalized abundances were used as input. (**B**) Heatmap based on normalized abundance indicating the top25 differentially expressed features in the WT and KO proteomes. KO3 not included for visualization purposes. The complete heatmap is provided in Supplementary Figure S4A. (**C**) MA plot of differentially regulated phosphosites. The log-fold change is plotted against the average log-intensity for all identified phosphorylation sites. (**D**) Volcano plot showing the differences between WT and KO from the integrated proteome and phosphoproteome. (**E**) Functional enrichment visualization of the biological processes enhanced in the over-representation analysis (ORA). (**F**) STRING network of the T-cell activation-related genes identified by Gene Set Enrichment Analysis (GSEA). Data from one experiment (WT, *n* = 4, and KO, *n* = 4, biological replicates).

We next performed a proteomics analysis on bulk splenic T-cells isolated from WT and KO mice to investigate the proteomic profile. As phosphorylation is a highly dominant process in T-cells, phospho-enrichment and phospho-integration analysis were included in the workflow. When assessing the general proteome, a distinct clustering pattern was observed between WT and KO animals (Figure 5A). Approximately 6000 proteins were identified, of which about 300 showed differential expression between the 2 genotypes (Figure 5B and Supplementary Figure S4A). The observed difference in the proteome was also reflected in the phosphoproteome, where nearly 25% of the phosphorylation sites were different in KO animals in comparison to WT (Figure 5C and Supplementary Figure S4B). Furthermore, the integration of the proteome and phospho-analysis showed a distinct phosphorylation pattern among the identified proteins, indicating that not only do protein expression levels differ, but also their phosphorylation profiles (Figure 5D).

When looking at the overrepresentation analysis of the biological processes, the top ten enriched pathways were related to activation, differentiation, and regulation of lymphocytes, which is consistent with our previous results in vivo and in vitro (Figure 5E). As T-cell activation was repeatedly seen in the analysis, we decided to examine the network of the T-cell activation-related proteins (Figure 5F). As expected, CD3^+^, CD4^+^ and CD8^+^ populations were decreased in the KO cells. Interestingly, we found that proteins related to T-cell survival, such as GIMAP1, BCL11b, and FAS, and T-cell proliferation like CD28, were reduced in the KO mice. These findings are supported by the low T-cell numbers in the KO mice. Notably, RhoH, a protein associated with efficient β-selection and thymocyte maturation, was significantly less abundant in the KO group. RhoH-deficient mice are characterized by impaired TCR selection and deficiency in T-cells, which closely resembles the phenotype observed in PTPN23-KO animals [20]. However, given the bulk nature of the proteomic analysis in our study, whether this reflects altered abundance within individual T-cell states or differences in cellular composition cannot be determined. Conversely, the upregulated proteins were mainly associated with a pro-inflammatory profile and differentiation of naïve T-cells into Th1, such as IL18R, TBX21, and SEMA4a, which was in line with our previous results. Particularly, we observed increased CD44 and CD74 expression, markers of effector and activated effector T-cells.

Taken together, these data showed a descriptive molecular signature of PTPN23 loss associated with substantial alterations in the proteomic and phosphoproteomic profiles of splenic T-cells, including proteins associated with T-cell proliferation, survival, differentiation, and inflammatory responses. However, because the bulk T-cell populations differ in their cellular composition between genotypes, these changes may reflect both altered T-cell composition and cell-intrinsic molecular effects of PTPN23 deficiency rather than purely subset-specific signaling changes.

### 3.5. T-Cell Development Is Altered upon PTPN23 Absence

Overall, our findings indicate that deletion of PTPN23 alters T-cell development and TCRαβ/γδ composition, with an increased representation of TCRγδ cells and a reduction in naïve T-cells. To verify these results, we performed scRNA-seq on thymocytes from WT and KO mice at an early age (four weeks of age).

**Figure 6:**
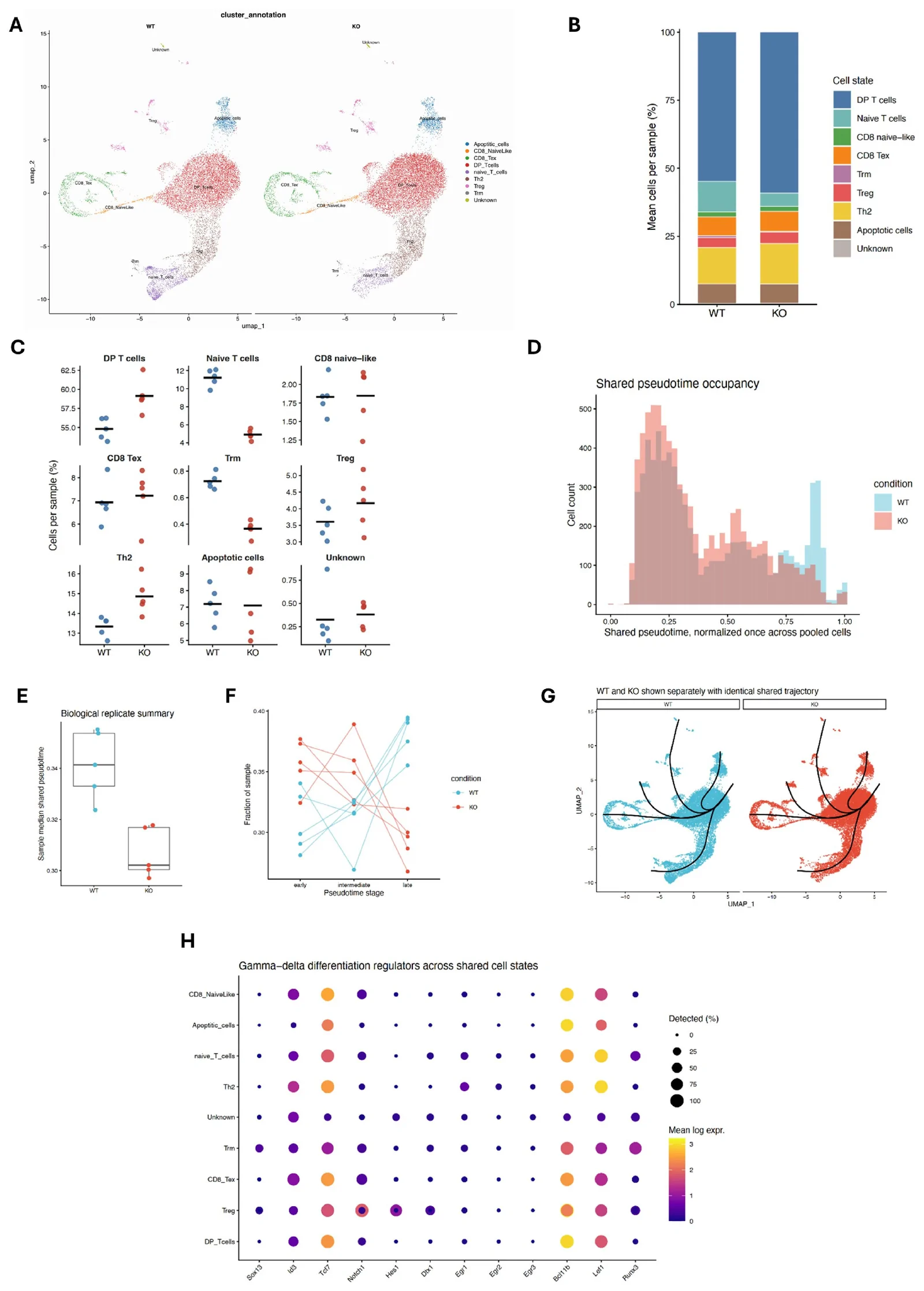
scRNA-seq on PTPN23-deleted mice revealed an accumulation of T-cells at early developmental stages. (**A**) UMAP visualization of annotated T-cell populations in WT (blue) and PTPN23-KO (red) mice, shown separately by genotype. (**B**) Mean cell-state composition per biological sample in WT and KO mice. (**C**) Sample-level comparison of the proportion of cells assigned to each cell state in WT and KO mice. (**D**) Distribution of cells along the shared pseudotime trajectory, normalized across pooled WT and KO cells. (**E**) Biological replicate summary of median shared pseudotime for WT and KO samples. (**F**) Sample-level occupancy of early, intermediate, and late pseudotime stages in WT and KO mice. (**G**) UMAP visualization of WT and KO cells shown separately with the identical shared developmental trajectory overlaid. (**H**) Dot plot showing expression of γδ T-cell differentiation regulators across shared cell states. Dot size indicates the percentage of cells expressing each gene, and color indicates mean log expression. Data are from one experiment (WT, *n* = 4, and KO, *n* = 4, biological replicates).

When examining cell clustering, we detected the same repertoire of cell populations in both genotypes, except for the naïve T-cell, where we found that, consistently with our previous experiments, the population was markedly reduced in the KO group compared to the WT (Figure 6A–C). This finding is consistent with altered representation of naïve T-cell states in PTPN23-deficient mice.

Consequently, we analyzed the scRNA-seq data using a genotype-independent pooled trajectory workflow (Figure 6D–F). We embedded, clustered, and analyzed WT and KO cells together, and applied CytoTRACE2 once to the pooled dataset. We selected a common root without using genotype labels by combining CytoTRACE2 with biological marker evidence. Among annotated DP_Tcells clusters, cluster 0 had the highest median CytoTRACE2 relative score and expressed developmental markers including Rag1/Rag2, Tcf7/Lef1, Bcl11b, Cd4, and Cd8. We, therefore, used this cluster as the shared root for Slingshot (Supplementary Figure S5A).

We then ran Slingshot once on the pooled embedding and inferred five lineages from the common DP_Tcells root. In the revised case, the same trajectory is shown on the pooled UMAP, overlaid on cells colored by genotype, and displayed separately for WT and KO using the identical trajectory overlay (Figure 6G). This analysis directly addresses the previous concern that WT and KO trajectories had been inferred separately. PTPN23-KO thymocytes showed a significant shift toward earlier T-cell developmental states. KO samples had a higher proportion of double-positive (DP) T-cells than WT (59.15% vs. 54.83%; BH-adjusted *p* = 0.0274) and fewer naïve T-cells (4.91% vs. 11.21%; BH-adjusted *p* = 0.0274). Consistently, KO samples had significantly lower pseudotime and reduced late-stage representation (BH-adjusted *p* = 0.0203 for both). Together, these results indicate that PTPN23 loss is associated with accumulation of early-stage thymocytes and reduced progression toward later T-cell developmental states. Flow cytometry independently confirmed an accumulation of DN thymocytes, providing additional support for altered representation of early thymocyte developmental states (Supplementary Figure S5B).

We next examined regulators implicated in γδ and αβ T-cell differentiation. Cell-state dot plots and UMAP expression maps showed that Tcf7, Id3, Bcl11b, Lef1, Egr-family genes, and Runx-family genes were expressed in state-dependent patterns across the shared thymocyte embedding (Figure 6H and Supplementary Figure S5). Sox13, Hes1, and Dtx1 were detectable but relatively sparse and were, therefore, interpreted cautiously. Genes including Sox13, Id3, Tcf7, Notch1, Dtx1, Egr1, Egr2, Egr3, Bcl11b, Lef1, and Runx-family genes showed significant expression dynamics along the main shared trajectory in focused tradeSeq analysis. Pathway/module score plots for Notch, ERK/EGR, TCR signaling, gamma-delta-associated, and alpha-beta-associated programs also varied along pseudotime, consistent with developmental-state-dependent regulatory programs. However, sample-aware within-cell-state comparisons of individual regulator expression and pathway module scores did not identify robust genotype-associated differences after multiple-testing correction. Thus, the regulatory analysis provides biological context for the inferred developmental states but does not support a strong claim that PTPN23 deficiency directly activates or suppresses a specific gamma-delta differentiation pathway at the transcriptional level.

## 4. Discussion

Many members of the PTP family are expressed in T-cells, where they play essential roles in regulating TCR signaling and maintaining the phosphatase–kinase balance [21,22]. Among them, PTPN2 has been shown to regulate early T-cell lineage commitment by attenuating STAT5 signaling, thereby influencing αβ versus γδ T-cell development through modulation of LCK and STAT5 pathways [23]. Interestingly, deletion of PTPN2 in intestinal epithelial cells was associated with increased expression of PTPN23 and altered regulation of IL-18, a cytokine involved in both innate and adaptive immune responses [24,25]. PTPN23 was not only shown to mediate ESCRT-associated receptor trafficking but also to regulate immune-associated pathways in the intestine, such as IgA transcytosis [11]. Together, these observations suggest that, in addition to its established role in intracellular receptor trafficking, PTPN23 may participate in the regulation of immune-cell functions. Our findings extend these observations by identifying a previously unrecognized role for PTPN23 in T-cell development and differentiation. Unlike phosphatases such as PTPN2, which have established roles in modulating TCR-associated signaling pathways, the potential contribution of PTPN23 to T-cell development has remained largely unexplored. Our study, therefore, provides new evidence linking PTPN23-dependent thymocyte maturation and signaling regulation to T-cell homeostasis and differentiation.

The results showed that mice lacking PTPN23 in T-cells exhibited an age-dependent phenotype characterized by a significant reduction of CD3^+^ cells. The remaining T-cells displayed an increased production of inflammatory mediators, along with an increased number of TCRγδ cells, particularly in mucosal tissues. Notably, the number of naïve T-cells decreased in KO mice, while the frequencies of central and effector T-cell populations were higher compared to WT mice.

The mechanisms underlying the αβ versus γδ T-cell lineage decision are not yet fully understood. The signal-strength model proposes that differences in the strength and duration of TCR signaling contribute to T-cell fate, with stronger and/or sustained signals favoring γδ lineage development, whereas weaker signals can promote αβ lineage selection. TCRγδ signaling has been associated with stronger downstream MAPK signaling, including prolonged activation of ERK and the early growth response (Egr) transcription factors. Enhanced ERK signaling has been proposed to support γδ lineage development by promoting a non-canonical signaling program that engages a distinct set of proteins involved in TCRγδ commitment [26]. Differences in the abundance or activity of these downstream signaling molecules may, therefore, contribute to the feedback mechanisms that regulate TCR signal strength and influence lineage commitment.

ERK is a downstream effector of several signaling pathways, including those activated by receptor tyrosine kinases, such as EGFR. Although EGFR expression is predominantly associated with epithelial cells, it has also been detected in several immune cell populations [27]. EGFR internalization is mediated, at least in part, through the ESCRT pathway, in which PTPN23 plays an important role [28]. Given the established role of PTPN23 in ESCRT-associated receptor trafficking, we speculate that altered trafficking or turnover of signaling receptors represents one potential mechanism through which PTPN23 deficiency could affect signaling strength and, consequently, T-cell differentiation. Such alterations could potentially influence signaling pathways involved in the αβ versus γδ lineage decision. However, the present study does not directly establish alterations in receptor internalization, recycling, or downstream ERK signaling in T-cells. Therefore, these mechanisms remain hypothetical and will require further experimental investigation to determine whether altered receptor trafficking and TCR-associated signaling contribute to the T-cell differentiation and inflammatory phenotypes observed in PTPN23-deficient mice.

Interestingly, the observed age-dependent effect may be attributed to the age-associated changes of the γδ T-cell population. γδ T-cells undergo age-dependent developmental and functional changes, which may contribute to the more pronounced mucosal phenotype observed in older KO animals and explain the differences observed between young and adult mice in our study [29].

Apart from its potential role in T-cell fate, we showed that PTPN23 may also influence T-cell cytokine production and the differentiation from naïve to effector cells. Upon antigen activation, naïve T-cells proliferate, differentiate into effector T-cells, and migrate to the site of inflammation, where they later become memory T-cells [30]. The proteomic analysis in our study showed increased levels of proteins associated with Th1 differentiation and CD44 expression, although the specific cell population responsible for these changes could not be determined due to the study design. We hypothesized that PTPN23 may affect pathways involved in the differentiation of naïve T-cells into effector cells, which could contribute to the enhanced cytokine production observed in PTPN23-deficient T-cells [31]. Further functional studies are required to validate these findings.

The d-shared-trajectory analysis showed that PTPN23-deficient thymocytes are enriched at earlier developmental states and have reduced late-stage representation, supporting altered developmental-state occupancy rather than an artifact of separately scaled pseudotime trajectories. Because pseudotime is inferred from static scRNA-seq, these findings do not demonstrate a physical developmental block but are consistent with reduced progression toward later T-cell developmental states.

γδ T-cell differentiation is regulated by transcriptional programs involving SOX13, TCF1/LEF1, and ID3, while TCR signal strength can influence αβ/γδ lineage choice through ERK–EGR–ID3 signaling [32,33,34,35]. In our data, these regulators varied along pseudotime, but no robust within-state genotype-associated differences were detected after accounting for biological replicates and cell-state composition. Thus, the predominant scRNA-seq phenotype of PTPN23 loss is altered developmental-state occupancy rather than a strong transcriptional shift within matched states.

Importantly, the CD4-Cre promoter used in this transgenic mouse line is active mainly at the DP stage and, therefore, our model cannot distinguish definitively whether the observed phenotype results primarily from altered thymocyte maturation, lineage commitment, differential survival of T-cell subsets, or a combination of these processes. To address this limitation, future studies using Cre models with earlier or stage-specific activity, such as appropriate Lck-Cre models, would be useful to determine whether PTPN23 contributes to the development of thymocyte progenitors and to define more precisely the developmental stage at which PTPN23 exerts its function in T-cells [36]. These alternative models would help to decipher the specific immune molecular processes in which PTPN23 is involved.

Taken together, our findings indicate that T-cell-specific PTPN23 deficiency is associated with marked alterations in T-cell abundance, TCRαβ/γδ composition, developmental-state representation, and cytokine profiles. These findings provide a framework for further investigation of the mechanisms by which PTPN23 may contribute to T-cell development and immune homeostasis.

### Limitations

This study has several limitations that should be considered when interpreting the findings. Overall, an insufficient T-cell number from KO mice, either in lymphoid organs or mucosal organs, prevented sorting into individual subpopulations. Particularly, the number of immune cells from mucosal organs in certain experiments was very low, which prevented further downstream analyses. Second, during in vitro experiments, the viability yield after T-cell activation was relatively low (20–50%), particularly at 48 h, which may have affected the accuracy and consistency of cytokine readouts, and therefore, the results should be interpreted carefully. In the proteomic approach, the initial sample input contained the bulk T-cell population. Therefore, we cannot exclude the possibility that the changes observed were due to the different T-cell composition of the initial input. Additionally, proximal TCR signaling and receptor trafficking were not directly assessed; therefore, the potential involvement of these pathways remains hypothetical, as stated in the discussion.

The use of the CD4-Cre model resulted in PTPN23 deletion during thymic development, after the earliest DN stages, which made it difficult to fully distinguish whether the observed phenotype was primarily caused by impaired thymocyte development, altered peripheral T-cell homeostasis, or a combination of both. Although our different analyses identified several pathways associated with T-cell differentiation and activation, the precise molecular mechanisms downstream of PTPN23 were not functionally validated. Furthermore, the use of the CD4-Cre model limited our ability to determine whether PTPN23 plays a role in the earliest stages of thymocyte development, as Cre-mediated deletion occurred only after the earliest DN stages. Therefore, the contribution of PTPN23 to the development or survival of earlier thymic progenitors cannot be ignored.

Despite these limitations, our results provide evidence that PTPN23 deficiency is associated with altered T-cell development, immune homeostasis, TCRαβ/γδ composition, and cytokine profiles.

## Data Availability

The data generated in this study are publicly available. Proteomic data have been deposited in Zenodo and are available at https://doi.org/10.5281/zenodo.20131605. Single-cell RNA-sequencing data have been deposited in the NCBI BioProject database under accession number PRJNA1464504.

## Supplementary Materials

The following supporting information can be downloaded at: https://www.mdpi.com/article/10.3390/cells15181694/s1, Figure S1: T-cells from KO mice show tissue-specific downregulation of PTPN23. Figure S2: PTPN23-deficient mice exhibited reduced spleen size in adulthood. Figure S3: Effector T cells were present at higher frequencies in KO mice, whereas their total numbers were unchanged. Figure S4: Proteomic analysis revealed different protein and phosphorylation profile in KO compared to WT. Figure S5: Expression dynamics of T-cell developmental regulators along the shared pseudotime trajectory in WT and PTPN23 KO cells. Table S1: Sex distribution. Table S2: Chemicals reagents, probes, and recombinant proteins. Table S3: Antibodies.

## Abbreviations

ACK	ammonium chloride potassium
DP	double-positive
DN	double-negative
EGFR	epidermal growth factor receptor
Egr	early growth response
ESCRT	endosomal sorting complex required for transport
IHC	immunohistochemistry
KO	knock-out
LPL	lamina propria
MALT	mucosa-associated lymphoid tissue
MEICs	murine endoscopic index of colitis severity
MHC	major histocompatibility complex
mLN	mesenteric lymph nodes
PMA	phorbol-myristate acetate
PTPN2	PTP non-receptor type 2
PTPN23	PTP non-receptor type 23
PTPs	protein tyrosine phosphatases
scRNA-seq	single-cell RNA sequencing
SP	single-positive
TMT	tandem mass tag
TCR	T-cell receptor
UMI	unique molecular identifier
WT	wild-type
