# Supplementary material for: T-Cell Specific Deletion of PTPN23 Alters T-Cell Development and TCRαβ/γδ Composition
Source: Cells. 2026 Sep 18;15(18):1694. doi: 10.3390/cells15181694 (PMC13605098; doi:10.3390/cells15181694)
Supplement: Supplementary file 1 [file cells-15-01694-s001.zip › cells-4540276-supplementary.pdf]

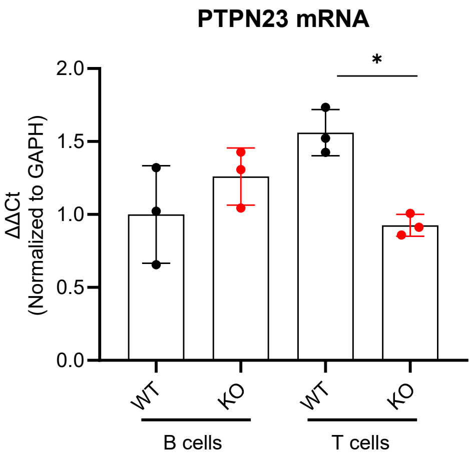

Supplementary Figure S1. T-cells from KO mice show tissue-specific downregulation of PTPN23. Normalized mRNA expression of PTPN23 in T cells and B cells from WT (black) and KO (red) mice. *p*-values were determined by ordinary one-way ANOVA. *$p < 0.05$. Unmarked comparisons are not statistically significant. Error bars represent mean ± SD.

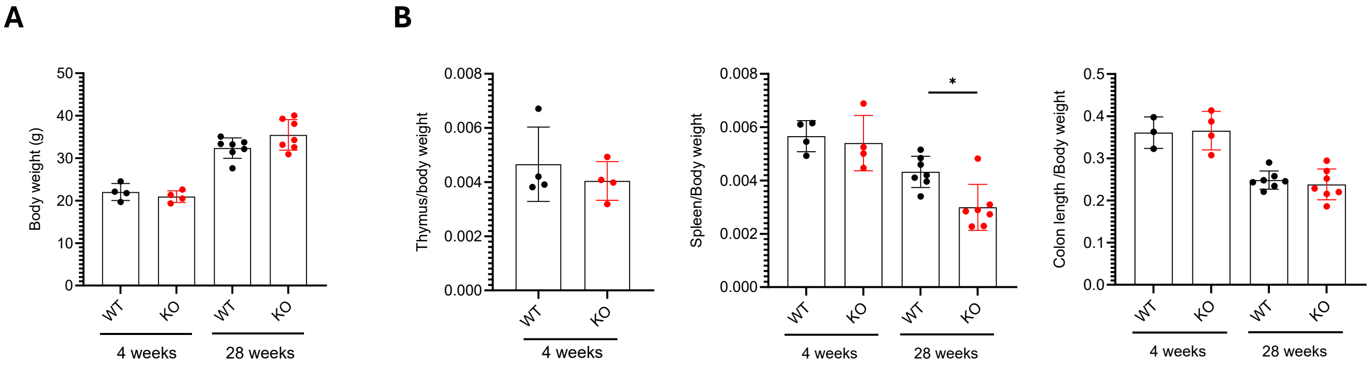

Supplementary Figure S2. PTPN23-deficient mice exhibited reduced spleen size in adulthood. (**A)** Body weight in WT and KO mice at young and adult ages. (**B)** Normalized thymus weight, spleen weight, and colon length in WT and KO mice. Data from one experiment consisting of WT group (*n*=4-7) and KO group (*n*=4-7) for each aging group. *p*-values were determined by the 2-tailed Mann-Whitney U Test; **p* < 0.05. Unmarked comparisons are not statistically significant. Error bars represent mean ± SD.

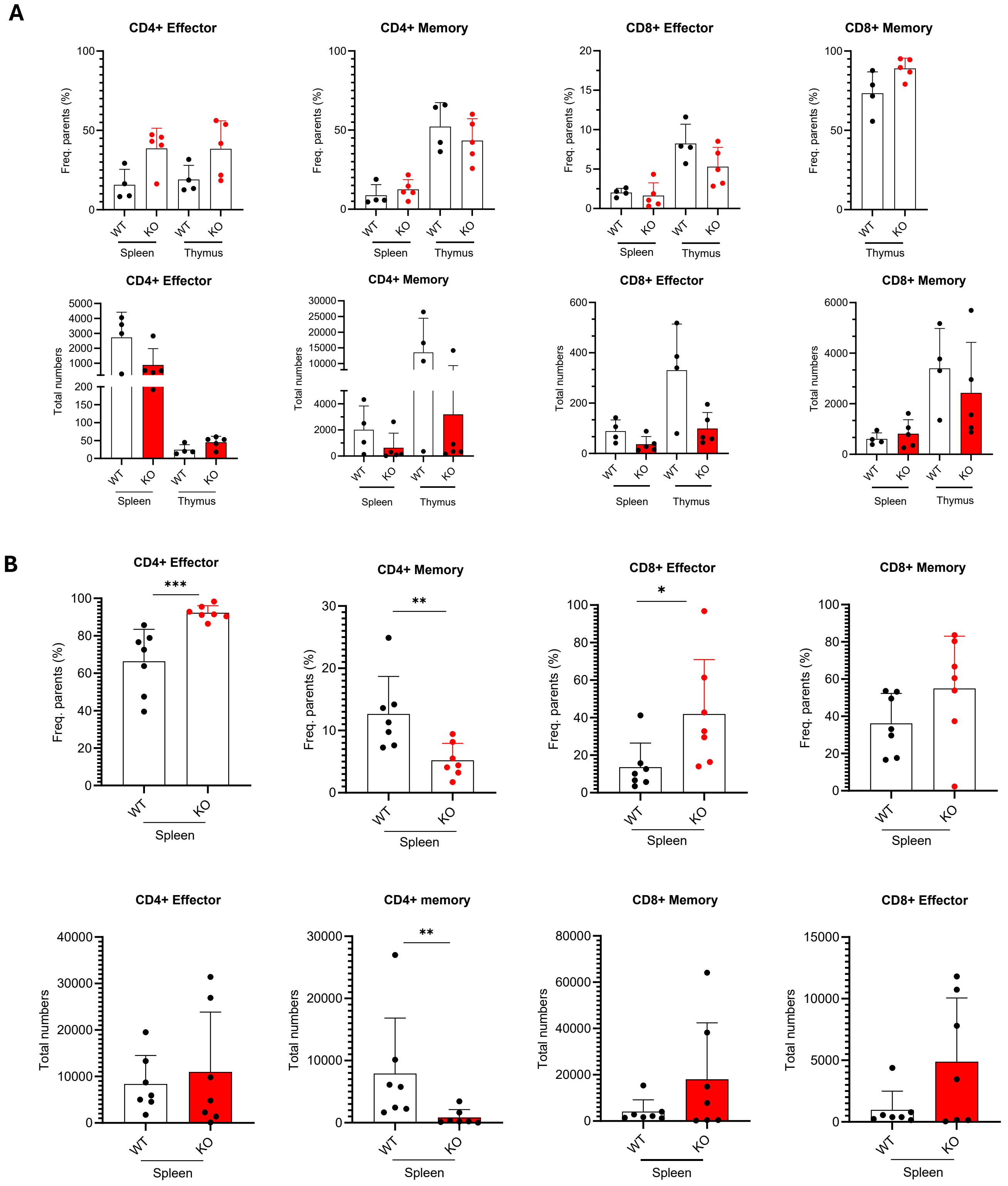

Supplementary Figure S3. Effector T cells were present at higher frequencies in KO mice, whereas their total numbers were unchanged. (**A**). Frequencies of parents and total numbers of effector and memory CD4⁺ T cells and CD8⁺ T cells from lymphoid organs from young WT and KO mice. (**B**). Frequencies of parents and total numbers of effector and memory CD4⁺ T cells and CD8⁺ T cells from lymphoid organs from adult WT and KO mice. Data from one experiment consisting of WT group (*n*=4-7) and KO group (*n*=4-7) for each aging group. *p* values were determined by 2-tailed Mann-Whitney U Test; *= *p*<0.05, ****p*<0.001. Unmarked comparisons are not statistically significant. Error bars represent mean ± SD.

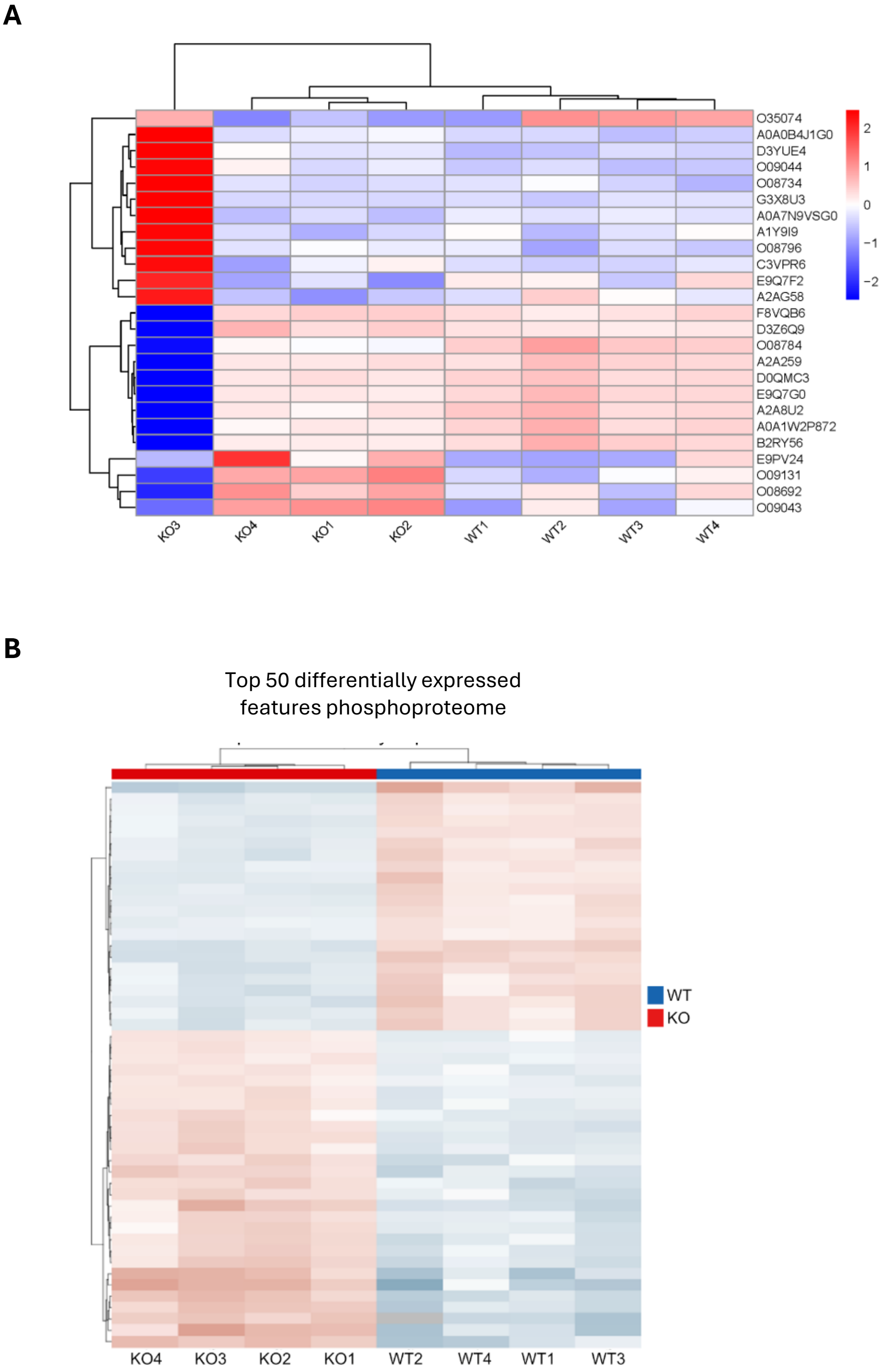

Supplementary Figure S4. Proteomic analysis revealed different protein and phosphorylation profile in KO compared to WT. **(A)** Heatmap based on normalized abundance indicating top25 differentially expressed features in the WT and KO proteomes. **(B)** Heatmap indicating the top 50 differentially expressed phosphorylation sites in the WT and KO mice.

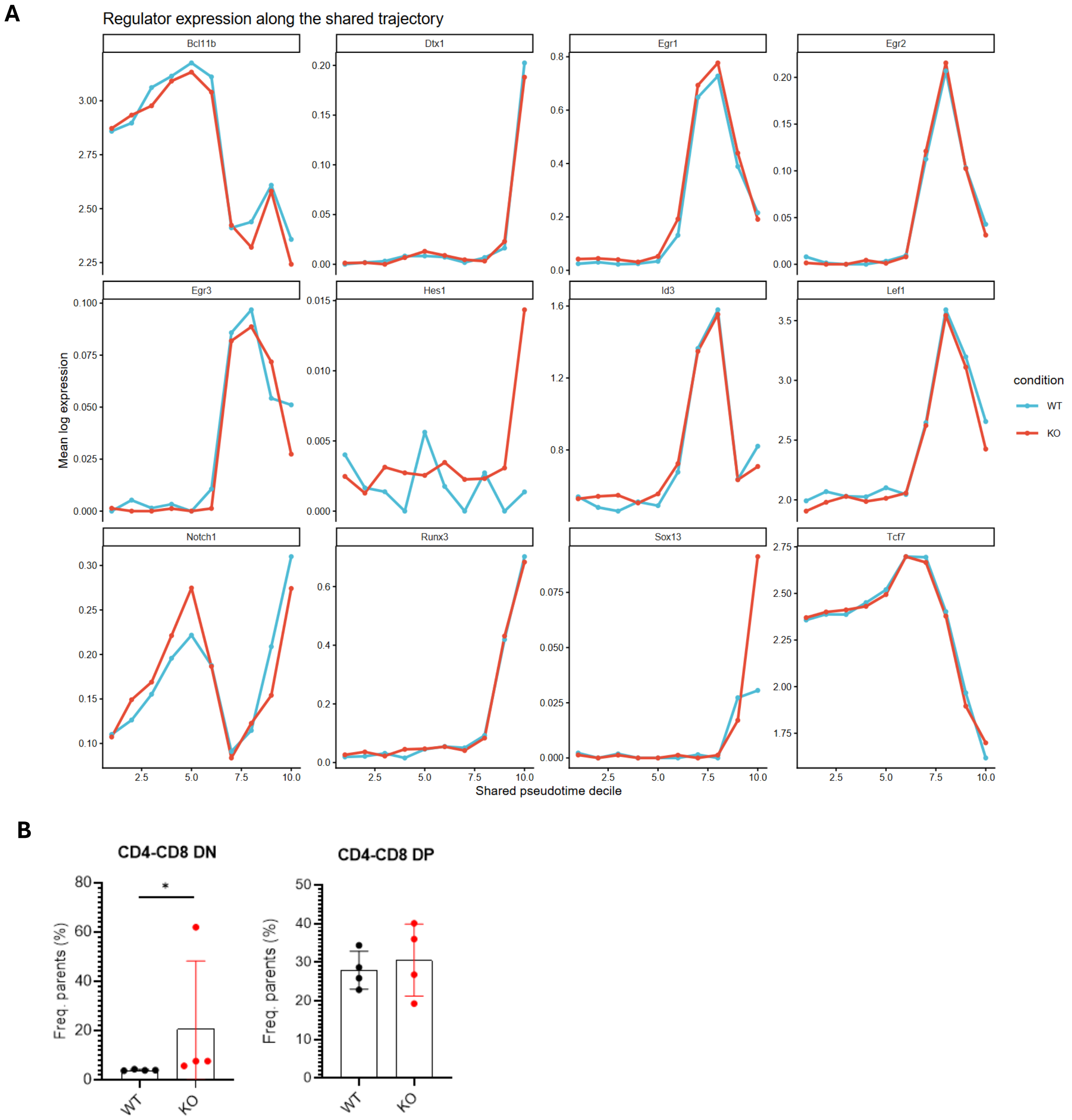

Supplementary Figure S5. Expression dynamics of T-cell developmental regulators along the shared pseudotime trajectory in WT and PTPN23 KO cells. **(A)** Mean log expression of Bcl11b, Dtx1, Egr1, Egr2, Egr3, Hes1, Id3, Lef1, Notch1, Runx3, Sox13, and Tcf7 across shared pseudotime deciles in WT (blue) and PTPN23 KO (red) cells. The plots illustrate changes in developmental-regulator expression along the inferred T-cell trajectory and allow comparison of their expression dynamics between genotypes. **(B)** Flow data showing double-negative (DN; CD4-CD8-) and double-positive (DP; CD4+CD8+) cells from WT and KO thymus. Experiment consisting of WT group (*n*=4) and KO group (*n*=4). Age of the mice at the termination day was 4 weeks. Experiment consisting of WT group (*n*=4) and KO group (*n*=4). Age of the mice at the termination day was 8 weeks. P values were determined by 2-tailed Mann-Whitney U Test; *$p < 0.05$.

Supplementary Table S1. Sex distribution.

Figure	Sex	Genotype	Experiment ID
Figure 1	male	wt	1
	male	wt	2
	male	wt	3
	male	wt	4
	male	wt	5
	male	ko	6
	male	ko	7
	male	ko	8
	male	ko	9
	male	ko	10
	male	ko	11
	male	ko	12
Figure 2	male	wt	1
	female	wt	2
	male	wt	3
	male	wt	4
	male	ko	5
	male	ko	6
	male	ko	7
	male	ko	8
	female	ko	9
Figure 3	female	ko	1
	female	ko	2
	female	ko	3
	female	ko	4
	female	ko	5
	male	ko	6
	male	ko	7
	female	wt	8
	female	wt	9
	female	wt	10
	male	wt	11
	male	wt	12
	male	wt	13
	male	wt	14
Figure 4	male	wt	1
	male	wt	2
	male	wt	3
	female	wt	4
	male	ko	5
	male	ko	6
	female	ko	7
	female	ko	8
Figure 5	female	wt	1
	male	wt	2
	male	wt	3
	male	wt	4
	female	ko	5
	female	ko	6
	male	ko	7
	male	ko	8
Figure 6	female	wt	1
	female	wt	2
	female	wt	3
	female	wt	4
	female	wt	5
	female	ko	6
	female	ko	7
	female	ko	8
	female	ko	9
	female	ko	10

Supplementary Table S2. Chemicals reagents, probes, and recombinant proteins.			
	**Source**	**Identifier**	**Concentration**
**Ammoniumchlorid min. 99,5 %, Ph.Eur.**	P726.1	Karl Roth	
**Brefeldin A**	Sigma Aldrich / Merck	B6542-25MG	1:10000
**EasySep™ Mouse T-Cell Isolation Kit**	StemCell	19581	
**Histoclear**	Brunschwig	HS-200	
**Ionomycin**	Sigma Aldrich / Merck	I3909	1:750
**MEM Non-Essential Amino Acids Solution (100X)**	Sigma-Aldrich	M7145	1%
**NaPy 100 mM Sodium Pyruvate**	Thermo Fisher Scientific	11360-039	1%
**Percoll**	Sigma Aldrich / Merck	GE17-0891-02	30%
**Pertex**	Biosystems Switzerland AG	41-4012-00	
**Phorbol-12-myristate-13-acetate (PMA)**	Sigma Aldrich / Merck	P1585	1:20000
**RPMI**	Thermo Fisher Scientific	21875-034	
**Zombie NIR Fixable Viability Kit**	BioLegend	423106	1:500

Supplementary Table S3. Antibodies.

	Source	Identifier	Application	Dilution
**Hamster anti- CD80 (16-10A1) – BUV737**	BD Becton Dickinson Pharminge Biosciences	612773	FACS	100
**Hamster anti mouse CD69 (H1.2F3) – BV711**	Lucerna / BioLegend	104537	FACS	100
**Hamster anti mouse CTLA4 (CD152) – PE/Cy7**	Lucerna-Chem / BioLegend	106313	FACS	200
**Hamster anti-CD11c (N418) – eFluor450**	Thermo Fisher Scientific	48-0114-82	FACS	200
**Hamster anti-CTLA4 (CD152) - PE/Cy7**	BioLegend	106313	FACS	200
**Hamster anti-mouse CD28**	BD Becton Dickinson Pharminge	557393	Coating	2 µg/ml
**Hamster anti-mouse CD3a**	BD Becton Dickinson Pharminge	557306	Coating	10 µg/ml
**Mouse anti-CD8 – PE-CF594**	BD Becton Dickinson Pharminge Biosciences	562283	FACS	200
**Mouse anti-Granzyme B – PerCP-Cy5.5**	BioLegend	372211	FACS	100
**Mouse anti-NK1.1 (PK136) - BV711**	BioLegend	108745	FACS	100
**Rat anti- FoxP3 – AF488**	eBioscience	53-5773	FACS	100
**Rat anti- IFNg (XMG1.2) - PE/Cy7**	Lucerna-Chem / BioLegend	505826	FACS	100
**Rat anti- IL17A – BV510**	Lucerna-Chem / BioLegend	506933	FACS	100
**Rat anti mouse CD25 – PE**	BD Pharmingen	553866	FACS	100
**Rat anti mouse CD279 (PD1)**	Lucerna-Chem / BioLegend	135210	FACS	100
**Rat anti mouse CD3 (17A2) – BV785**	Lucerna-Chem / BioLegend	100232	FACS	200
**Rat anti mouse CD4 (RM4-5) – BV650**	BD Becton Dickinson Pharminge Biosciences	562283	FACS	200
**Rat anti mouse CD44 (IM7) - FITC**	Thermo Fisher Scientific / eBioscience	11-0441-85	FACS	200
**Rat anti mouse CD45 (30-F11) – BV480**	BD Becton Dickinson Pharminge Biosciences	566095	FACS	200
**Rat anti mouse CD62L (MEL 14) – BV605**	Lucerna / BioLegend	104437	FACS	200
**Rat anti mouse CD8 – PECF594**	BD Becton Dickinson Pharminge Biosciences	562283	FACS	200
**Rat anti mouse-B220 – PE-Cy5**	BD Pharmingen	552772	FACS	200
**Rat anti-CD11b (M1/70) – BV605**	Lucerna-Chem / BioLegend	101257	FACS	200
**Rat anti-CD25 – APC**	eBioscience	17-0251-81	FACS	100
**Rat anti-CD279 (PD1) (29F.1A12) - APC**	BioLegend	135210	FACS	100
**Rat anti-CD45 (30-F11) – BV480**	BD Becton Dickinson Pharminge Biosciences	566095	FACS	400
**Rat anti-CD45R/B220 (RA3-6B2) – BV570**	Lucerna-Chem / BioLegend	103237	FACS	200
**Rat anti-CD62L - BV605**	BD Becton Dickinson Pharminge Biosciences	563252	FACS	200
**Rat anti-F480 (BM8) – PE-Cy5**	Lucerna-Chem / BioLegend	123112	FACS	200
**Rat anti-IL12 – APC**	BioLegend	505206	FACS	100
**Rat anti-MHCII – AF700**	Lucerna-Chem / BioLegend	107622	FACS	100
